# Glycitein alleviates inflammation and apoptosis in keratinocytes via ROS-associated PI3K–Akt signalling pathway

**DOI:** 10.1515/biol-2025-1162

**Published:** 2025-09-17

**Authors:** Wenqian Sun, Jinyu Chen, LiHong He, Yating Chen

**Affiliations:** Department of Dermatology, The Affiliated Hospital of QingDao BinHai University, Qingdao, Shandong, 266404, China; Department of Dermatology, Ezhou Central Hospital, Ezhou, Hubei, 436000, China; Department of Outpatient, The General Hospital of Western Theater Command of Chinese People’s Liberation Army, Chengdu, Sichuan, 610000, China; Department of Skin Burn, The People’s Hospital of Kaizhou District. CQ, Chongqing, 405400, China

**Keywords:** glycitein, inflammation, ROS, PI3K, Akt signalling pathway, psoriasis

## Abstract

A chronic inflammatory skin disorder, psoriasis, affects 2–3% of people worldwide. A bioactive substance, glycitein (GCN), has several pharmacological characteristics. This work aims to evaluate the effects of GCN on the *in vitro* proliferation and death of human HaCaT keratinocytes. An *in vitro* model was created to simulate psoriatic features utilizing HaCaT keratinocytes activated by M5 cytokines. The 3-[4,5-dimethylthiazol-2-yl]-2,5-diphenyltetrazolium bromide test was used to quantify cell viability, whereas the BrdU assay was used to assess the proliferation rate. Using a DCFH-DA probe and an Annexin V-FITC/propidium iodide detection kit, flow cytometry was used to examine the generation of reactive oxygen species (ROS) and apoptosis, respectively. Western blot and quantitative polymerase chain reaction were employed to determine the amounts of phosphorylated Akt (p-Akt) and Akt proteins. GCN dramatically decreased the inflammation and hyperproliferation that cytokines caused in HaCaT keratinocytes. The alteration of mitochondrial membrane potential promoted apoptosis and caused cell cycle arrest at the sub-G1 phase, which indicates apoptotic DNA fragmentation. The suppression of the PI3K/Akt signalling pathway was linked to increased intracellular ROS levels brought on by GCN therapy. These results imply that GCN reduces inflammation and keratinocyte hyperproliferation by controlling cell cycle progression and apoptosis via ROS-associated inhibition of the PI3K/Akt pathway.

## Graphical abstract

**Figure:**
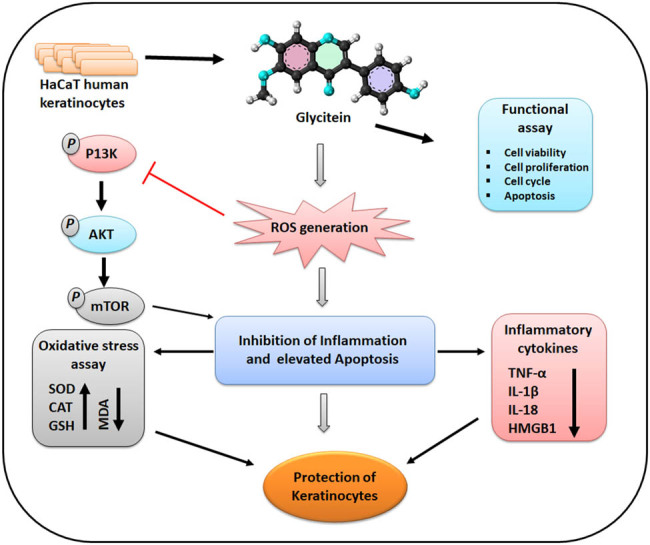

## 1 Introduction

Psoriasis is a persistent, non-contagious, erythematous scaly skin disorder that is defined by epidermal hyperplasia and inflammation. Genetics, spread of infection, allergies, metabolic problems, and autoimmunity are factors in its pathogenesis [1,2,3]. According to Gao et al. [4], it is a common chronic inflammatory illness with a growing frequency of 2–3% globally. Its defining characteristics are rapid proliferation, abnormal keratinocyte differentiation, and inflammatory cell infiltration into the epidermal layer and epidermis [4]. Although the precise pathophysiology of psoriasis is currently unknown, it is widely recognized that aberrant relationships among immune cells and keratinocytes are crucial to the disease’s progression [5]. The stimulating effect of keratinocytes by different cytokines released by immune cells in the lesion can cause hyperproliferation of keratinocytes, which consequently triggers the production of massive proinflammatory mediators by hyperproliferative keratinocytes to maintain or even intensify the inflammatory response [6,7]. Because psoriasis is difficult to treat and vulnerable to recurrence, research into novel therapies linked to keratinocyte proliferation and exacerbated inflammation is beneficial.

Reactive oxygen species (ROS) mediate apoptotic and inflammatory and other intracellular messenger pathways. Emerging evidence implicates ROS as a pivotal regulator of keratinocyte function in psoriasis, with elevated ROS levels promoting apoptosis while suppressing abnormal proliferation [8,9]. The cellular outcome depends on ROS concentration: low levels may promote aberrant proliferation, while higher levels can induce oxidative stress-mediated apoptosis [10,11]. Thus, modulating ROS levels to shift keratinocytes towards apoptosis represents a promising therapeutic avenue [12,13]. Apoptosis, a well-characterized form of programmed cell death, plays a vital role in maintaining tissue homeostasis, and its dysregulation is linked to several skin disorders [14,15]. Several studies have shown a functional connection between ROS and activation or suppression of key apoptotic regulators [16,17].

One of the most important pathways for surviving cells is the PI3K/Akt signalling pathway, a common autophagy-dependent transduction pathway that plays a role in vascular development, cell development, and various additional incorporates [18,19]. Related cytokines may promote PI3K in cells, activating downstream Akt and associated proteins and causing corresponding biological consequences. According to Guo et al. [20] and Mercurio et al. [21], this mechanism can control upward and downward associated targets in psoriasis, causing abnormal proliferation of epidermal cells. Mammalian target of rapamycin (mTOR) is aberrantly expressed downstream, whereas PTEN expression has decreased upstream. Psoriasis symptoms can be alleviated by suppressing endothelial growth factors by inhibition of the PI3K/Akt signalling pathway [22,23]. Recent findings indicate that elevated ROS may downregulate PI3K/Akt signalling, thereby promoting keratinocyte apoptosis and reducing inflammatory responses, suggesting a mechanistic link between oxidative stress and PI3K/Akt modulation in psoriasis. However, this interaction remains incompletely understood.

Bioflavonoids have been used in healthcare facilities subsequently because of their many biological properties, affordability, and excellent safety record [24]. In soybeans, glycitein (GCN), a 7,4′-dihydroxy-6-methoxyisofavone, is the third commonly encountered isofavone. According to Kim et al. [25], it is also commonly found in many cordyceps species of fungus. According to research, it exhibits anti-inflammatory [26], antioxidant [27], neurologically protective [28,29], liver-protective properties [30], and antitumor [31,32] effects. Despite its known pharmacological benefits, the role of GCN in psoriasis, particularly in regulating ROS-associated PI3K/Akt signalling, has not yet been investigated.

In this study, we employed M5 cytokine-stimulated HaCaT keratinocytes as an *in vitro* model of psoriasis to evaluate GCN’s antiproliferative and pro-apoptotic effects. We further explored GCN’s influence on the PI3K/Akt signalling pathway in the context of ROS generation, aiming to elucidate the molecular basis of GCN’s potential therapeutic action.

## 2 Materials and methods

### 2.1 Molecular docking analysis

The 3D structures of GCN were retrieved from the PubChem-NCBI database, while the target proteins Akt (PDB ID: 2UW9) and P13K (PDB ID: 6TNR) were obtained from the RCSB Protein Data Bank. The protein and ligand files were prepared by converting them into PDBQT format using Autodock Tools (v1.5.7 rc1) provided by the Scripps Research Institute. A docking grid was set up, and molecular docking simulations were conducted using Autodock Vina (v1.5.6, available at https://vina.scripps.edu). The docking results were further analysed using BIOVIA Discovery Studio Visualizer software to identify interactions.

### 2.2 Cell culture and *in vitro* model formation

Human HaCaT keratinocytes were acquired from the Chinese Academy of Science’s Kunming Cell Bank of Type Culture Collection (Kunming, China). Incubated at 37°C with 5% CO_2_, the cells were kept in Dulbecco’s modified eagle medium supplemented with 10% foetal bovine serum (FBS). By introducing M5 cocktail cytokines (IL-17A, IL-22, oncostatin M, IL-1α, and TNF-α) into the media of HaCaT keratinocytes at the final dosage of 2.5 ng/mL, a psoriasis-like keratinocyte type has been developed.

### 2.3 Cell viability and proliferation

The viability of HaCaT cells was evaluated using the 3-[4,5-dimethylthiazol-2-yl]-2,5-diphenyltetrazolium bromide (MTT) test. For 24 h, HaCaT cells (2 × 10^4^) were grown in 96-well plates. HaCaT cells were administered with 2.5 ng/mL of combined M5 cytokines and GCN (0, 5, 10, 20, 40, and 80 μM). After the maker’s instructions, the MTT reagent was utilized. At 490 nm, the absorbance density was determined by a microplate reader. Using the following formula, the cell viability ratio (%) was determined:

\[ \% \hspace{.5em}\text{viability}=(\text{test}\hspace{.5em}\text{sample}/\text{control})\times 100 \% .]\]

Although alternative assays such as cholecystokinin octapeptide (CCK-8) offer improved sensitivity and lower cytotoxicity, the MTT assay remains a robust method for endpoint viability analysis and was used here due to its reproducibility and accessibility in our setup.

### 2.4 Proliferation assessment using BrdU assay

Using the BrdU Cell Proliferation assessment kit (Santa Cruz Biotechnology), thymidine monobromodeoxyuridine (BrdU) was used to examine cell proliferation in settings involving *de novo*-synthesised DNA. In summary, 2 × 10^3^ HaCaT cells/well were scattered onto 96-well plates, and the cells were then incubated for 48 h at 37°C. After that, 20 mL of BrdU was included, and the mixture was incubated for 4 h at 37°C. One hundred millilitres of 3,3′,5,5′-tetramethylbenzidine peroxidase substrate was placed in the following mixture, which was incubated with anti-BrdU antibody and peroxidase-conjugated goat anti-mouse IgG for 30 min. The mixture was then left in the dark at ambient temperatures. Employing a microplate reader, the intensity of absorption at 450/550 nm was detected.

### 2.5 Assessment of superoxide dismutase (SOD) activity

A solution of 0.033 mM ethylenediamine tetraacetic acid, 3.3 mM methionine, 0.01 mM KCN, and 0.33 μg/mL riboflavin (Sigma, St. Louis, MO, USA) has been added to the supernatant. For 10 min, the cuvette holding the reaction solution was put in a box that was lit by a twenty-watt neon bulb. The quantity of SOD enzyme needed to inhibit the synthesis of chromogen (optical density at 560 nm) by 50% in 1 min under the assay circumstances is known as one unit of SOD function. This particular activity is quantified in units/min/mg protein.

### 2.6 Evaluation of glutathione (GSH) levels

The concentration of GSH was determined by interacting with 5,5′-dithiobis(2-nitrobenzoic) acid, which yielded 5′-thio (2-nitrobenzoic) acid (TNBA; Sigma, St. Louis, MO, USA), a yellow-colored molecule. A light spectrum of 412 nm was used to quantify the percentage of TNBA, and the quantity of GSH was expressed in nmol GSH/mg protein.

### 2.7 Catalase (CAT) enzyme assessment

After splitting H_2_O_2_ for 1 min, the CAT activity was ascertained. Dichromate/acetic acid reagent was used to halt the reaction, and the amount of H_2_O_2_ left behind for chromic acetate was determined at 570 nm. μmol/L H_2_O_2_ decomposed/min/mg protein was used to generate the CAT function.

### 2.8 Assessment of malondialdehyde (MDA) activity

Following the manufacturer’s recommendation, a lipid peroxidation MDA assay kit (Beyotime Institute of Biotechnology, Haimen, China) was employed to measure the MDA activity. GCN treatment (10 and 20 μM) was followed by the harvesting of HaCaT cells, lysing them with cell lysis buffer, and centrifuging them at 12,000 × *g* for 15 min at 4°C. After reacting with thiobarbituric acid in the resulting supernatant, the resulting reaction components were detected at 532 nm using a microplate reader. MDA level was adjusted, and the entire protein value was assessed using a BCA protein detection kit (Beyotime Institute of Biotechnology, Haimen, China).

### 2.9 Analysing apoptosis with fluorescence microscopy

Apoptotic and morphological modifications brought on by GCN were detected by acridine orange/ethidium bromide (AO/EB) and 4′,6-diamidino-2-phenylindole (DAPI) labelling. Essentially, GCN at 10 and 20 μM was added to HaCaT cells that were seeded in a 12-well plate the day before. Following a 2-h incubation period, cells were stimulated with M5, followed by a 48-h incubation period, 10 s of AO/EB (10 μg/mL each) staining, and two phosphate buffered saline (PBS) washes. For DAPI labelling, cells were permeabilized with 0.1% Triton X-100 and fixed with 4% paraformaldehyde. They were then stained with DAPI (1 μM) in PBS, and fluorescence microscopy (Nikon, Tokyo, Japan) images were taken at ×200 magnification.

### 2.10 Cell cycle analysis

HaCaT cells were cultivated, incubated with M5 for 48 h, and treated with GCN for 2 h. The cells were subsequently trypsinized, fixed in 70% ethanol, and kept at −20°C. Following a PBS wash, the cells were treated for 15 min with propidium iodide (PI) staining buffer before being exposed to flow cytometric analysis (BD C6 Accuri flow cytometry, USA).

### 2.11 JC-1 staining

Apoptosis is activated by ROS when ΔΨ*m* is lost. At high membrane potential, JC-1 reversibly forms “J aggregates” with red fluorescence, but when ΔΨ*m* is lost, it forms green fluorescence because of “J monomers.” The assay was carried out by seeding HaCaT cells in 12-well plates and stimulating them with M5 cytokines and GCN, respectively. Following 48 h of incubation, cells were stained for 30 min with 1 μM of JC-1 dye and then exposed to flow cytometry.

### 2.12 Apoptosis assay using flow cytometry

Flow cytometry was used to identify apoptosis. Annexin-V-FITC and PI labelling were used to quantitatively identify phosphatidylserine on the surface of apoptotic cells. Following 24-h incubation, with M5 cytokines and GCN for 2 h, the cells were harvested by centrifugation at 800 rpm for 5 min, followed by a PBS wash, another centrifugation, and resuspension in PBS. After 1 h of Annexin-V-FITC staining and 30 min of PI staining, the cells were examined using a FACScan system.

### 2.13 Assessment of intracellular ROS concentrations

A peroxide-sensitive fluorescent detector, DCFH-DA, was used to assess ROS levels within the cell following directions provided by the manufacturer. To summarize, HaCaT cells were grown in a six-well plate and stimulated with different doses of GCN and M5 cytokines for the specified durations. After 30 min at 37°C in medium without FBS, cells were treated with DCFH-DA at a final concentration of 10 μM. They were then rinsed three times with media. The ROS contents were measured using a flow cytometer.

### 2.14 Western blotting analysis

HaCaT keratinocytes were cultivated for 24 h after being seeded in six-well plates. Cells at 60–80% diameter were induced with M5 (5 ng/mL) for 30 min after being treated with GCN for 24 h. Cell lysis buffer containing 1 mM phenylmethylsulfonyl fluoride was used to harvest and lyse the cells for 20 min on ice. For 15 min, cell lysates were centrifuged at 14,000 rpm. The BCA kit (Pierce, USA) was used to measure the protein level in the final solution. After separating approximately 15 μg of protein using sodium dodecyl sulfate polyacrylamide gel electrophoresis, the protein was moved to polyvinylidene fluoride membranes and blocked for 2 h at ambient temperature with 5% nonfat milk. The primary antibodies (Akt, p-Akt, pPI3K, PI3K, mTOR) were then reared for the complete night at 4°C at a dilution of 1:1,000. Following three washes with TBS-T washing, the membranes were treated for 2 h at ambient temperature with HRP-conjugated secondary antibody. The membranes were then moved to the ChemiDoc™ XRS + System (Bio-Rad, USA) to visualize the protein signals after being treated with chemiluminescence substrate (Pierce, USA). ImageJ software was utilized to measure the protein bands. The internal reference was β-actin.

### 2.15 Enzyme-linked immunosorbent assay (ELISA)

GCN (10 and 20 μM) was administered to the M5-induced HaCaT cells for 24 h. Then, using an ELISA kit (Abcam Technology, Cambridge, UK) as directed by the manufacturer, inflammatory substances TNF-α (Cat No. ab181421), high mobility group box 1 (HMGB1) (Cat No. ab227731), IL-1β (Cat No. ab214025; Abcam, Cambridge, MA, USA), and IL-18 (Cat No. ab215539) in the supernatant were identified. In short, a 96-well plate coated with primary antibodies towards cytokines such as TNF-α, HMGB1, IL-18, and IL-1β was filled with 50 mL of typical samples and a control blank. HRP-conjugated detection antibodies (100 mL) were then incubated for 1 h at 37°C. Following five rounds of washing, substrates A and B were added for an additional 15 min of incubation. In a microplate reader, the absorbance was lastly measured at 450 nm. By creating a standard curve, the levels of cytokines in multiple samples were measured.

### 2.16 Docking of molecules

The interacting affinities of GCN for PI3K and Akt were evaluated using molecular docking. PubChem was able to determine the molecular structure of GCN (https://pubchem.ncbi.nlm.nih.gov/compound/5280805). PI3K and Akt’s three-dimensional structures were taken using the Protein Data Bank database (https://www.uniprot.org/). The binding sites among GCN, PI3K, and Akt have been determined using the AutoDock 4.2 software. PyMol 2.2 was used to visualize the outcomes of the docking process.

### 2.17 Assessment of the data

Each investigation was carried out with a minimum of three biological duplicates in triplicate. Standard error, mean, and median were displayed for the data. For statistical analysis, Prism 9 (GraphPad Software, USA) was used. Analyses of variance (ANOVA) with Dunnett’s post-hoc test were used to examine for variances across various groups. The *p*-value was deemed substantial provided it was below 0.05.

## 3 Results

To investigate whether GCN could regulate ROS levels, suppress inflammatory signalling, and modulate the PI3K/Akt pathway in psoriatic keratinocytes, we conducted a series of *in vitro* experiments using M5-stimulated HaCaT cells.

### 3.1 GCN impact on the viability of HaCaT keratinocytes

**Figure 1:**
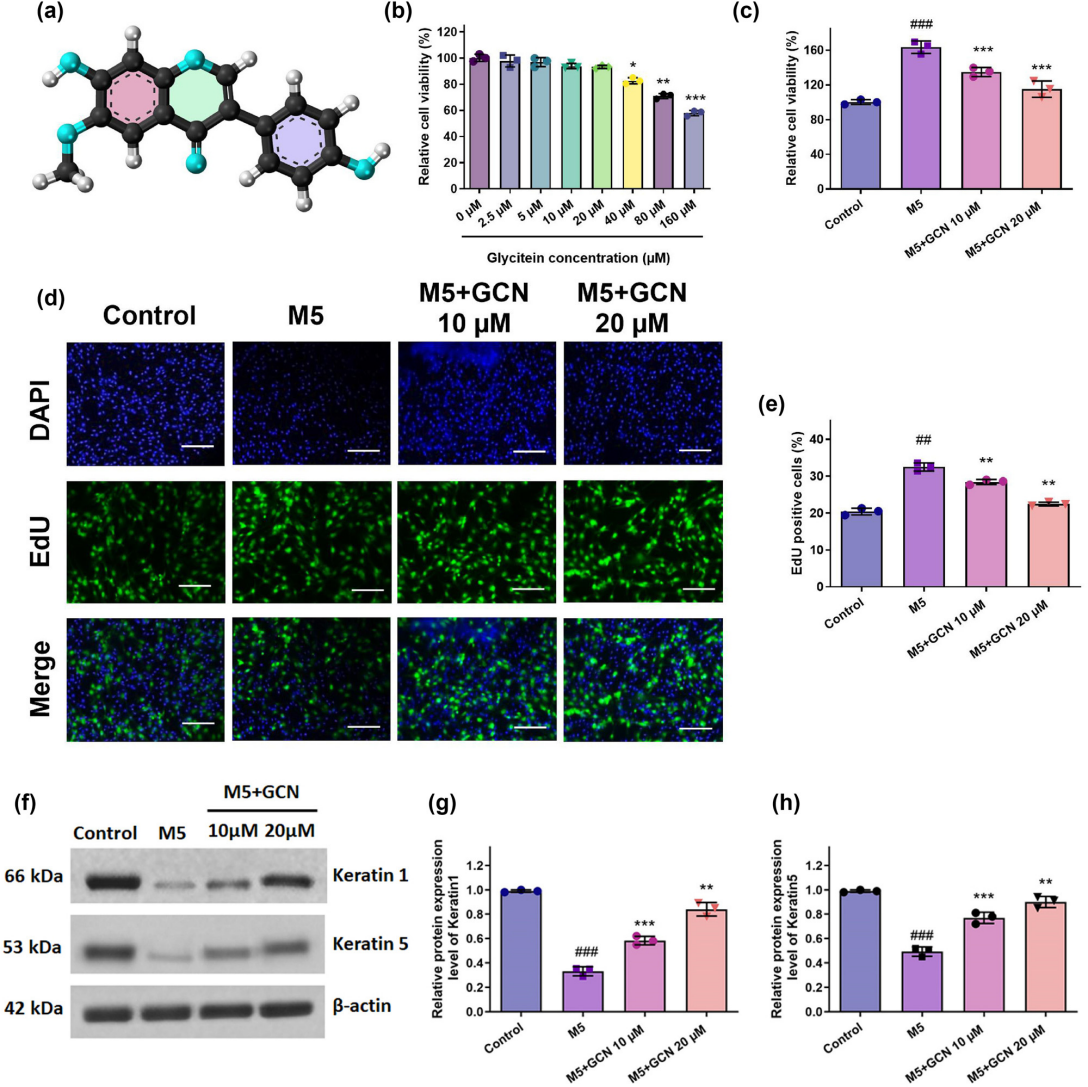
GCN impact on the growth of HaCaT cells proliferation after being activated with M5 cytokines. Cell viability (a) and (b) was assessed using the MTT test; (c) the proliferation rate was assessed using the BrdU assay; (d) the proportion of cells exhibiting BrdU-positive (BrdU^+^) staining was found. (e) and (f) The levels of KRT1 and KRT5 protein expression were investigated using a Western blot. (g) and (h) Shows band intensities assessed by Image J software evaluation to protein expression levels graphically. Compared to the control, ^###^ *p* < 0.05 comparing control, **p < 0.01, ***0.05 compared to the M5 group. SEM ± mean was used to express the data.

The chemical structure of GCN, a naturally occurring isoflavone having anti-inflammatory qualities, is displayed in Figure 1a. The MTT examination was employed to assess the cell viability of HaCaT keratinocytes subjected to GCN. Following a 48-h GCN administration, no discernible toxicity was seen at values lower than 40 μM. At doses greater than 80 μM, GCN dramatically reduced the cell viability of HaCaT keratinocytes (Figure 1b). Thus, for the following investigations, keratinocytes were exposed to 10–20 μM GCN.

### 3.2 GCN attenuates M5-induced hyperproliferation in HaCaT keratinocytes

The widely utilized psoriatic keratinocyte model was employed to create an *in vitro* model of mixed M5 cytokine-stimulated HaCaT keratinocytes to study the phenotypic characteristics of prevalent psoriasis. Cell viability was assessed using MTT to assess the impact of GCN therapy on M5-stimulated HaCaT keratinocytes. The outcome demonstrated that M5 stimulation greatly increased HaCaT keratinocyte proliferation. However, GCN at 10 and 20 μM reduced the hyperproliferation caused by M5 (Figure 1c). Furthermore, the BrdU staining experiment demonstrated that M5 therapy significantly increased HaCaT cell proliferation. Furthermore, in a dose-related approach, GCN substantially inhibited the proliferation of HaCaT cells caused by M5 in contrast to the untreated group (Figure 1d and e). Therefore, our data validate that M5 promoted cell proliferation, but GCN in administration inhibited this mechanism.

### 3.3 GCN facilitated the differentiation of HaCaT cells induced by M5

To further evaluate the impact of GCN on the differentiation potential of M5-stimulated HaCaT cells, we employed the Western blotting test to identify the keratinocyte differentiation markers Keratin1 and Keratin5. In comparison with untreated cells, findings demonstrated that M5-stimulated HaCaT cells had lower levels of Keratin1 and Keratin5 protein; however, these lower levels were largely restored after GCN treatment (Figure 1f–h). These outcomes exhibited that the abnormal differentiation of HaCaT cells caused by M5 may be mitigated by GCN.

### 3.4 GCN reduced keratinocyte inflammation brought on by M5

**Figure 2:**
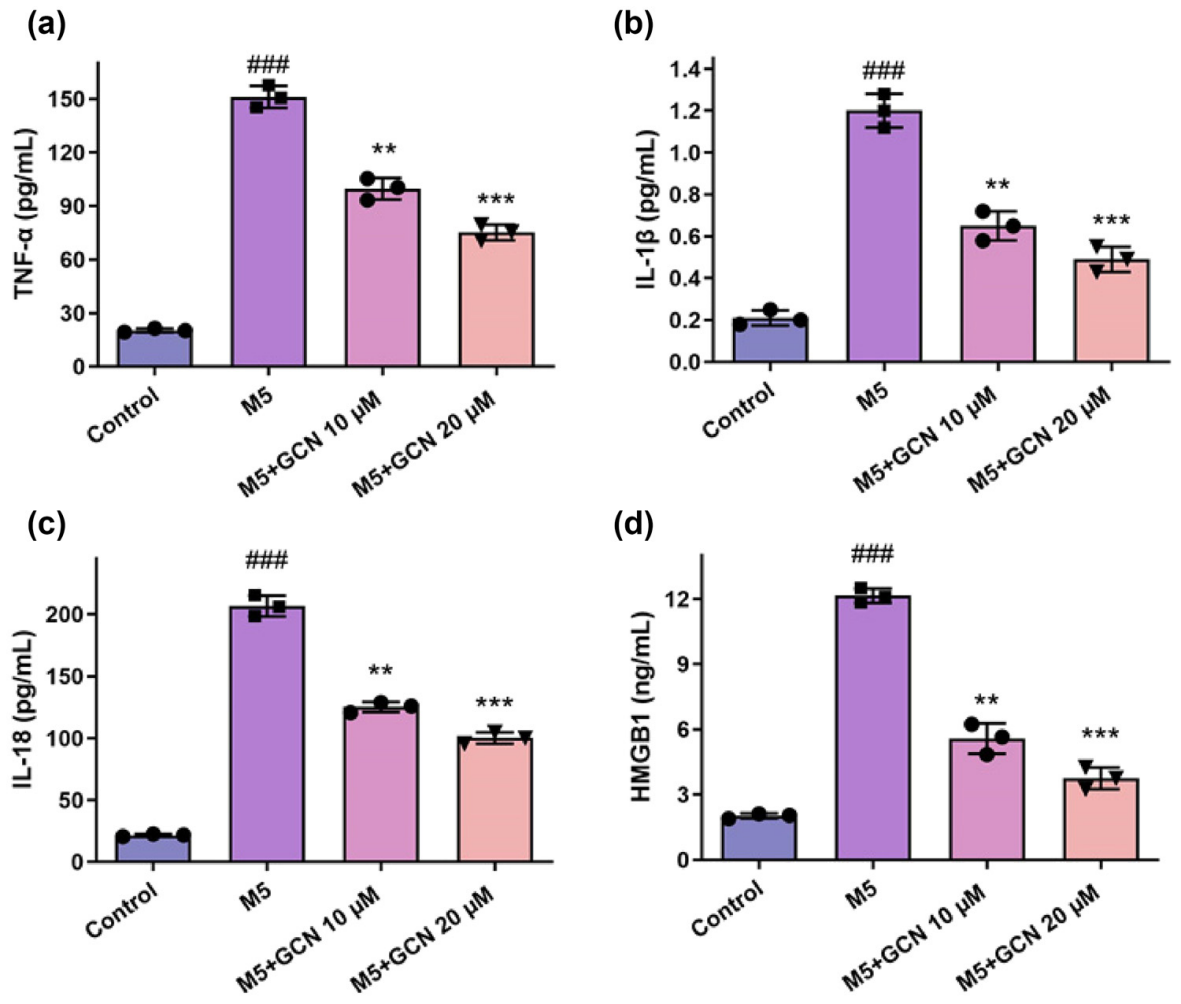
GCN attenuates M5-induced pro-inflammatory cytokine production in HaCaT cells. ELISA was used to measure the levels of inflammatory cytokines in cell culture supernatants. (a) TNF-α, (b) IL-1β, (c) IL-18, and (d) HMGB1 were significantly elevated following M5 treatment compared to the control group. GCN co-treatment (10 and 20 μM) significantly reduced these cytokine levels in a dose-dependent manner. Data are expressed as mean ± SEM (*n* = 4). ^###^ *p* < 0.001 vs control; **p < 0.01, ***p < 0.001 vs M5-treated group.

The pathophysiology of psoriasis is significantly influenced by proinflammatory cytokines. To investigate the impact of GCN on M5-induced a reaction of inflammation, more investigation was carried out on the components associated with inflammation following various therapies (Figure 2). Following M5 cytokine exposition, the expression levels of inflammation-related indicators (TNF-α, HMGB1, IL-18, and IL-1β) in HaCaT cells increased considerably, and following treatment with various doses of GCN, the release of TNF-α, HMGB1, IL-18, and IL-1β was repressed (Figure 2a–d). The findings showed that GCN reduced the M5-induced proinflammatory response’s inflammatory cytokine production.

### 3.5 GCN prevents oxidative stress in M5-stimulated keratinocytes

**Figure 3:**
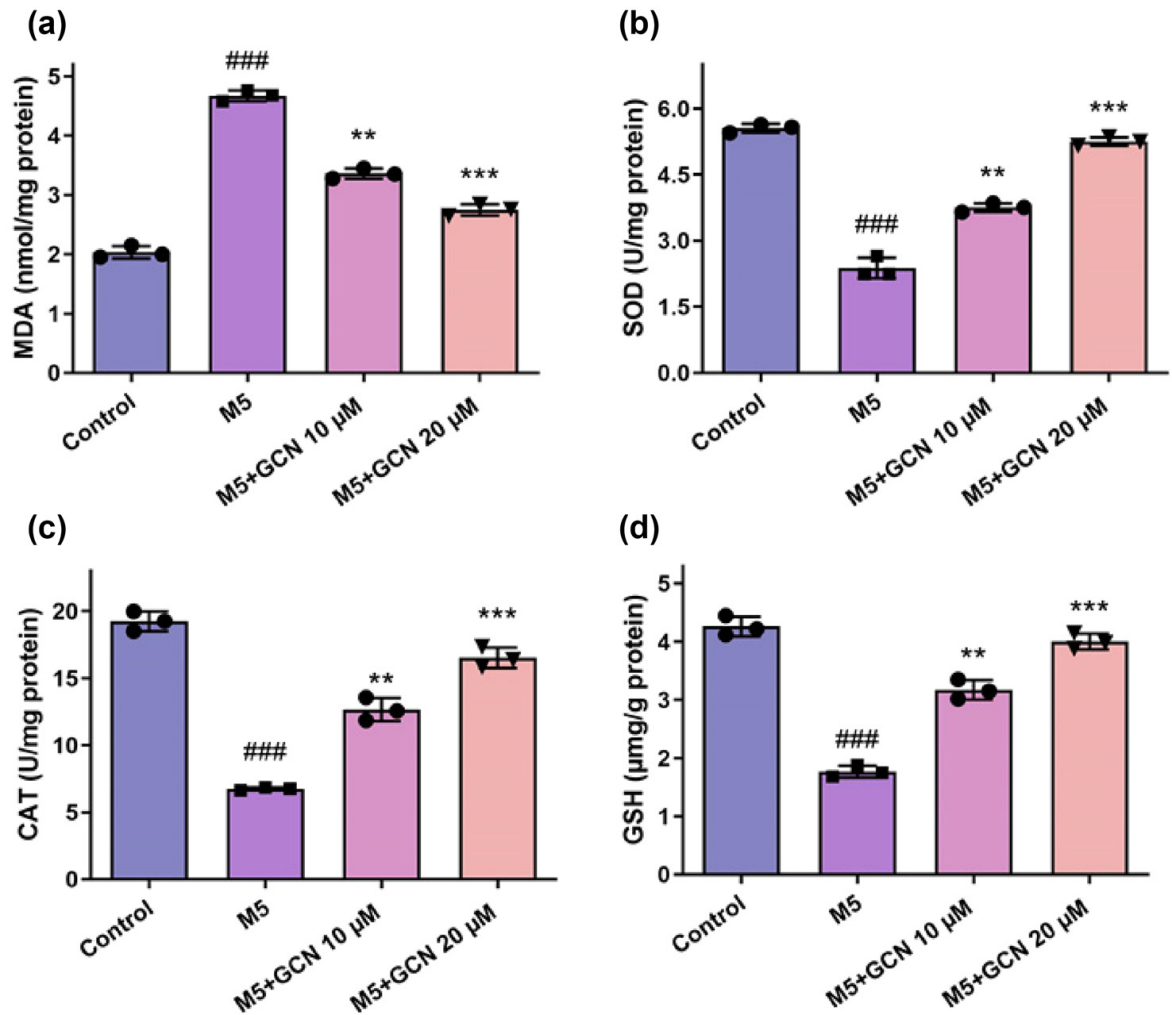
GCN prevented M5-stimulated HaCaT cells from experiencing oxidative damage. (a–d) GCN raised SOD, CAT, and GSH levels in a dose-related way while decreasing MDA levels. ^###^ *p* < 0.005 vs control; **p < 0.01 and ***p < 0.05 vs M5. Differential expression testing was performed with ANOVA.

Psoriasis is mostly caused by oxidative stress. As a result, an oxidative stress assay was implemented. The findings showed that M5 stimulated oxidative stress by increasing MDA while decreasing SOD, CAT, and GSH. Nevertheless, GCN administration dramatically reduced MDA levels while dose-dependently raising SOD, CAT, and GSH levels (Figure 3a–d). GCN as a whole prevented HaCaT cells from encountering oxidative stress.

### 3.6 GCN induces apoptosis to prevent keratinocyte proliferation

**Figure 4:**
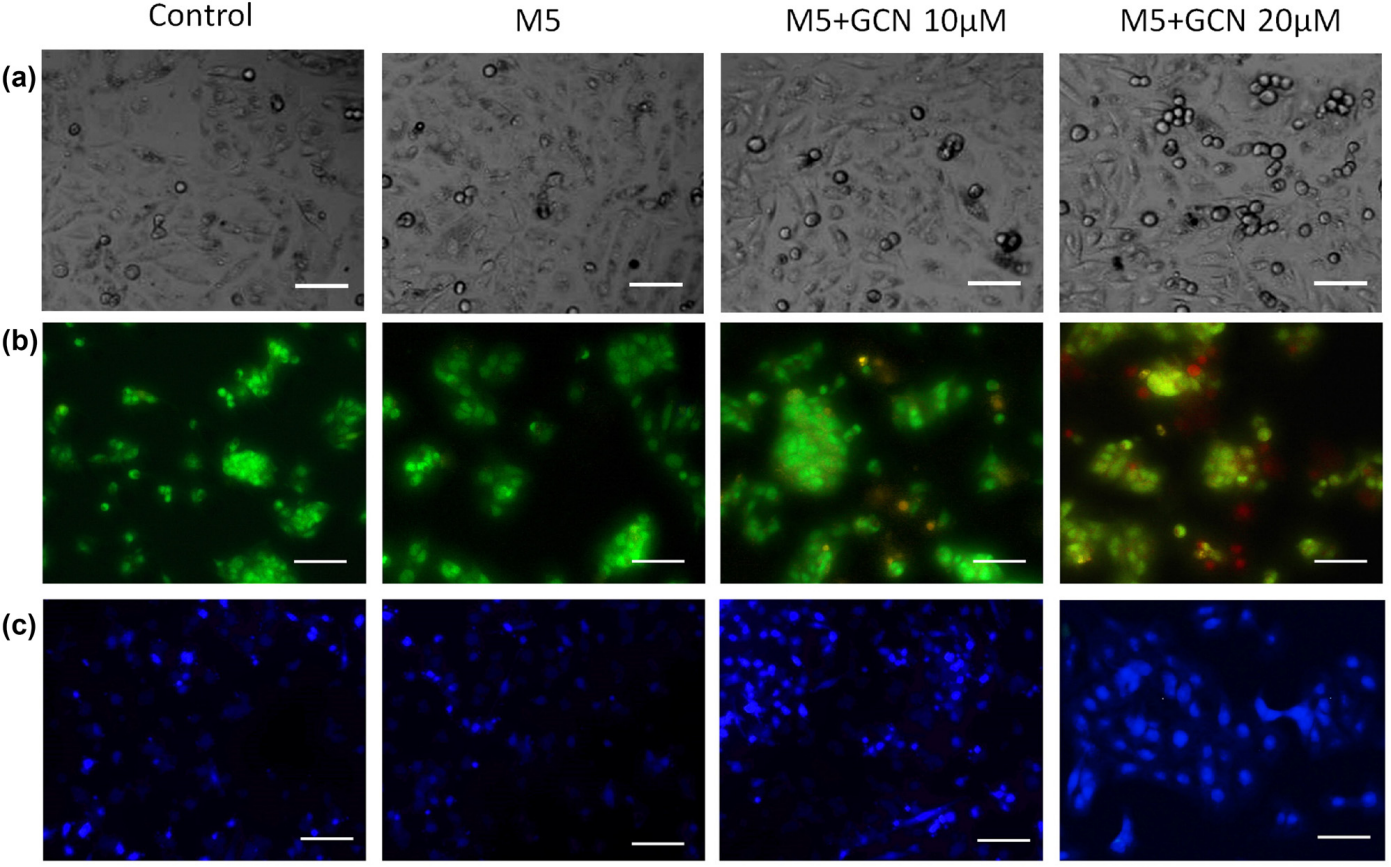
GCN inhibits the excessive proliferation of HaCaT cells and causes apoptosis. (a) Phase contrast microscopy visualization of morphological alterations caused by GCN. (b) AO/EB simultaneous staining shows apoptotic alterations brought on by GCN, which result in condensed chromatin and apoptotic structures. (c) Using DAPI labelling, nuclear and apoptotic alterations were observed. The fluorescence microscope was used to take the pictures at a magnification of ×200.

It was also discovered that GCN caused cell death by inducing apoptosis in the cells. At dosages of 10 and 20 μM, GCN-treated cells showed considerable morphological loss, including nuclear pyknosis, apoptotic blebs, and nucleus condensate formation as demonstrated by structural phase-contrast examination, AO/EtBr, and DAPI marking (Figure 4a–c).

### 3.7 GCN-induced ROS production disrupts mitochondrial membrane potential (ΔΨ*m*)

**Figure 5:**
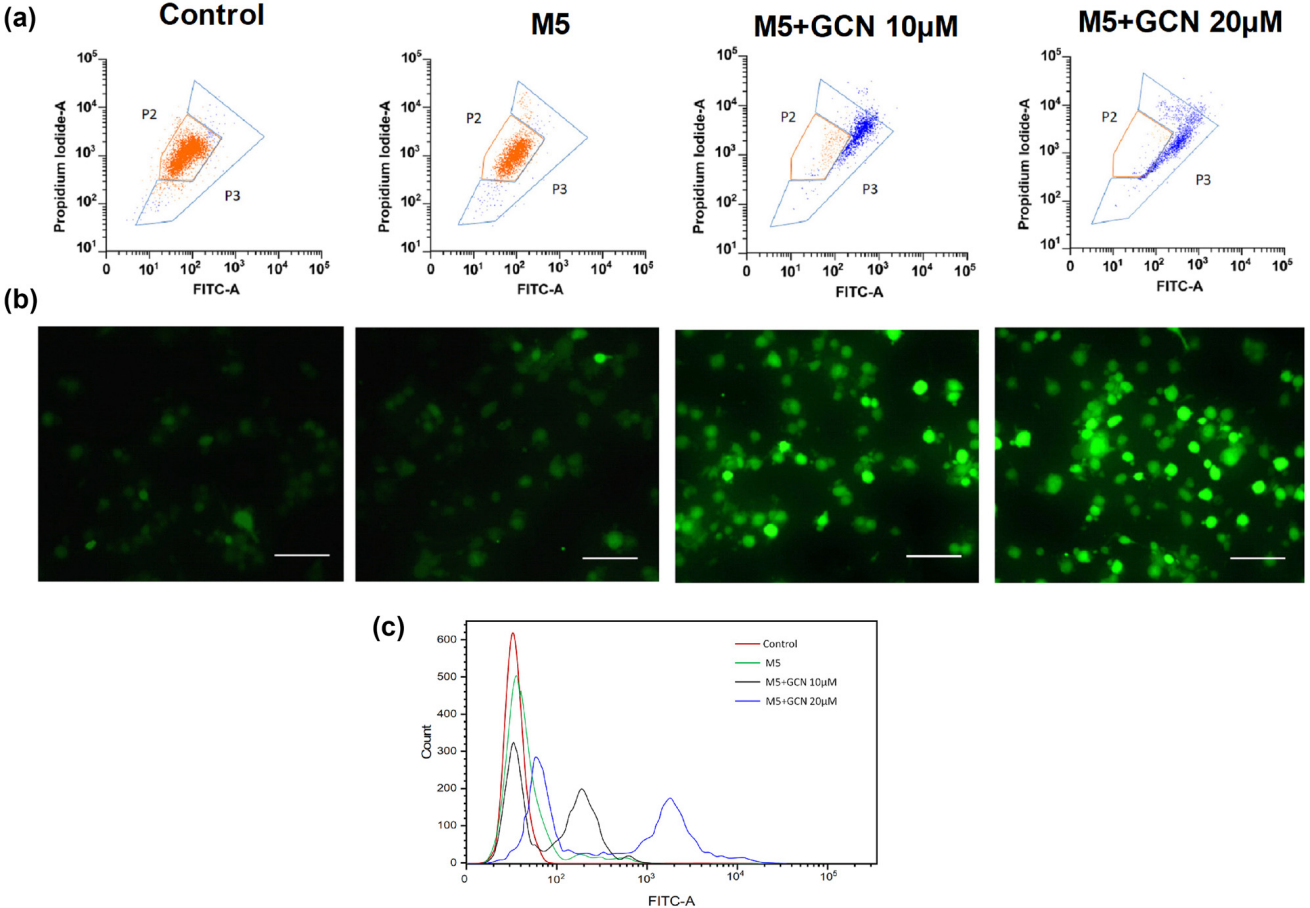
GCN induces human skin cells to experience oxidative damage and a decrease in mitochondrial membrane potential. (a) The oxidation and reduction capacity of the membrane of the mitochondria due to GCN was assessed using JC1 staining. (b) DCFDA probing was used to assess the ROS generated by GCN. At a magnification of ×200, the fluorescent pictures were taken. (c) The mean fluorescence intensity (MFI) was then measured and displayed as a bar graph.

Strong formation of ROS causes ΔΨ*m* to be disrupted and further triggers the apoptotic process. To examine the decrease of mitochondrial membrane potential (ΔΨ*m*) by M5, JC-1 labelling was used. GCN administration over 24 h caused the fluorescence to shift into green, suggesting a reduction in ΔΨ*m*. In contrast, J aggregates in control cells began to show orange fluorescence, whereas the JC-1 monomers, formed as a consequence of the loss of ΔΨ*m*, showed green fluorescence (Figure 5a).

### 3.8 GCN suppresses the generation of ROS in M5-induced HaCaT cells

The impact of GCN on aggregate intracellular ROS was then assessed using DCFDA labelling in M5-stimulated cells. GCN treatment at 10 and 20 μM substantially elevated ROS levels in cells, and a phenomenon of oxidative stress occurred, as shown in Figure 5b and c. The quantities of ROS generated were measured by flow cytometry, in which groups administered GCN demonstrated a shift of the peak closer to the right side, suggesting that GCN therapy elevated the generation of ROS in HaCaT keratinocytes.

### 3.9 GCN causes cell cycle arrest and apoptosis

**Figure 6:**
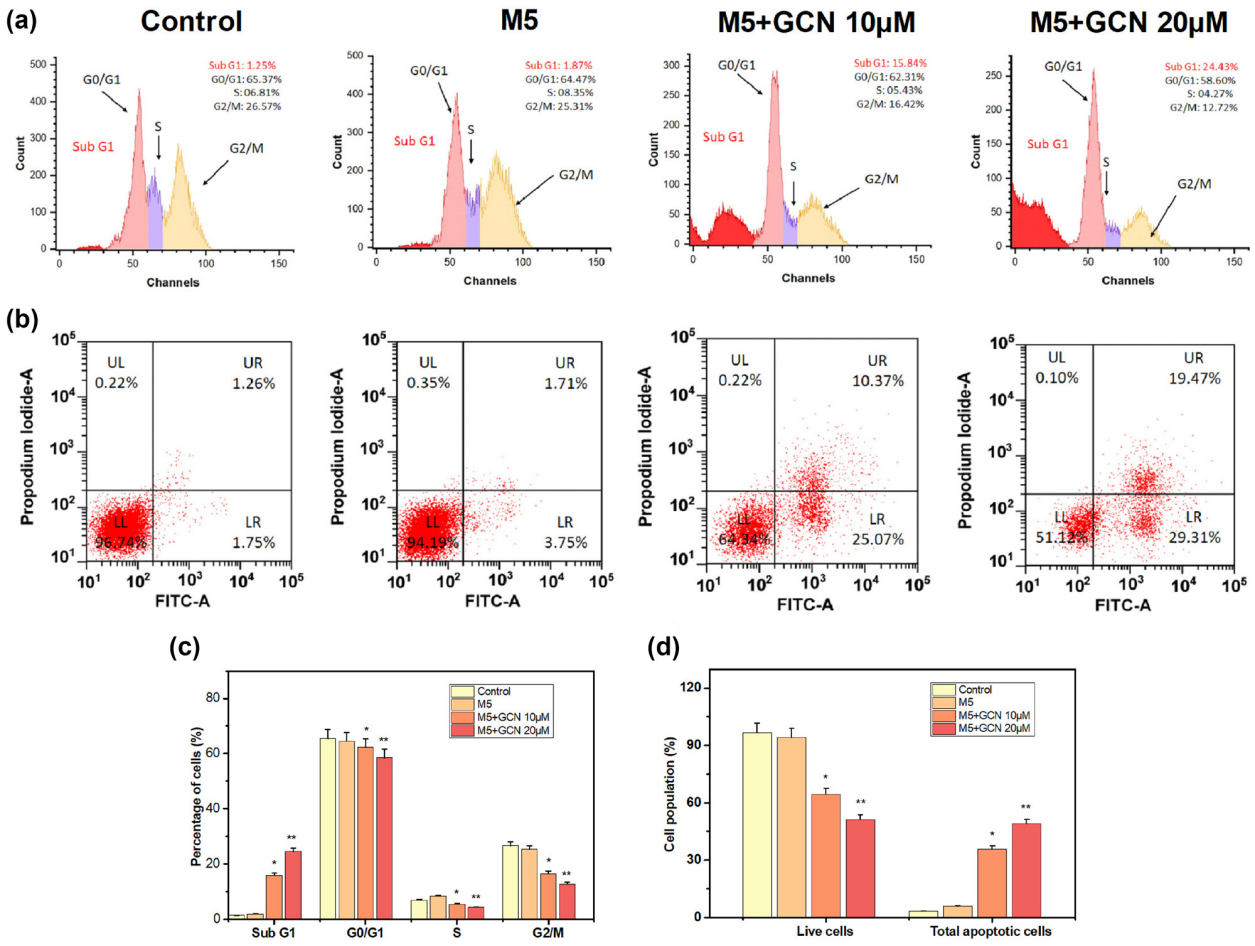
GCN causes keratinocytes to stop developing in the sub-G1 phase, which triggers earlier apoptosis. Using flow cytometric analysis, it was discovered that GCN induced cell cycle arrest and apoptosis. The spatial distribution of PI staining in several cell cycle phases is shown in (a), and the histograms’ maxima correspond to these phases. (b) After being labelled with Annexin V-FITC/PI, apoptotic cells were examined using flow cytometry. (c) A graph with bars was used to show the proportion of cells used in every stage. (d) The proportion of live cells and total apoptotic cells in every category was computed. Data are displayed as mean ± standard deviation. Prism 8 was used to calculate the *p*-values using a one-way ANOVA; *p < 0.01; **p < 0.05.

Integrating flow cytometry, the influence on the proportion of cell cycle phase scattering was studied. Sub-G1 phase arrest can be used to evaluate DNA fragmentation, which serves as a powerful indicator of the initiation of apoptosis. According to the flow cytometric evaluation used in this study, the level of GCN treatment elevated the DNA disintegration, which was identified by a distinct and sharp peak that represented the population of “Sub G1” (apoptotic cells). We further noticed that the deposits of cells in the Sub G1 peak rose more substantially at concentrations of 5 and 10 μM in comparison to control cells. The proportion of S and G2/M phases decreased in tandem with this (Figure 6a and c). PI and annexin V-FITC labelling were used in flow cytometry to assess the impact of GCN on the apoptosis of HaCaT keratinocytes. When compared to control cells, the proportion of early and late apoptosis was significantly elevated after 24 h of GCN (10 and 20 μM) treatment (Figure 6b and d).

### 3.10 Binding interaction of GCN with P13K and Akt

**Figure 7:**
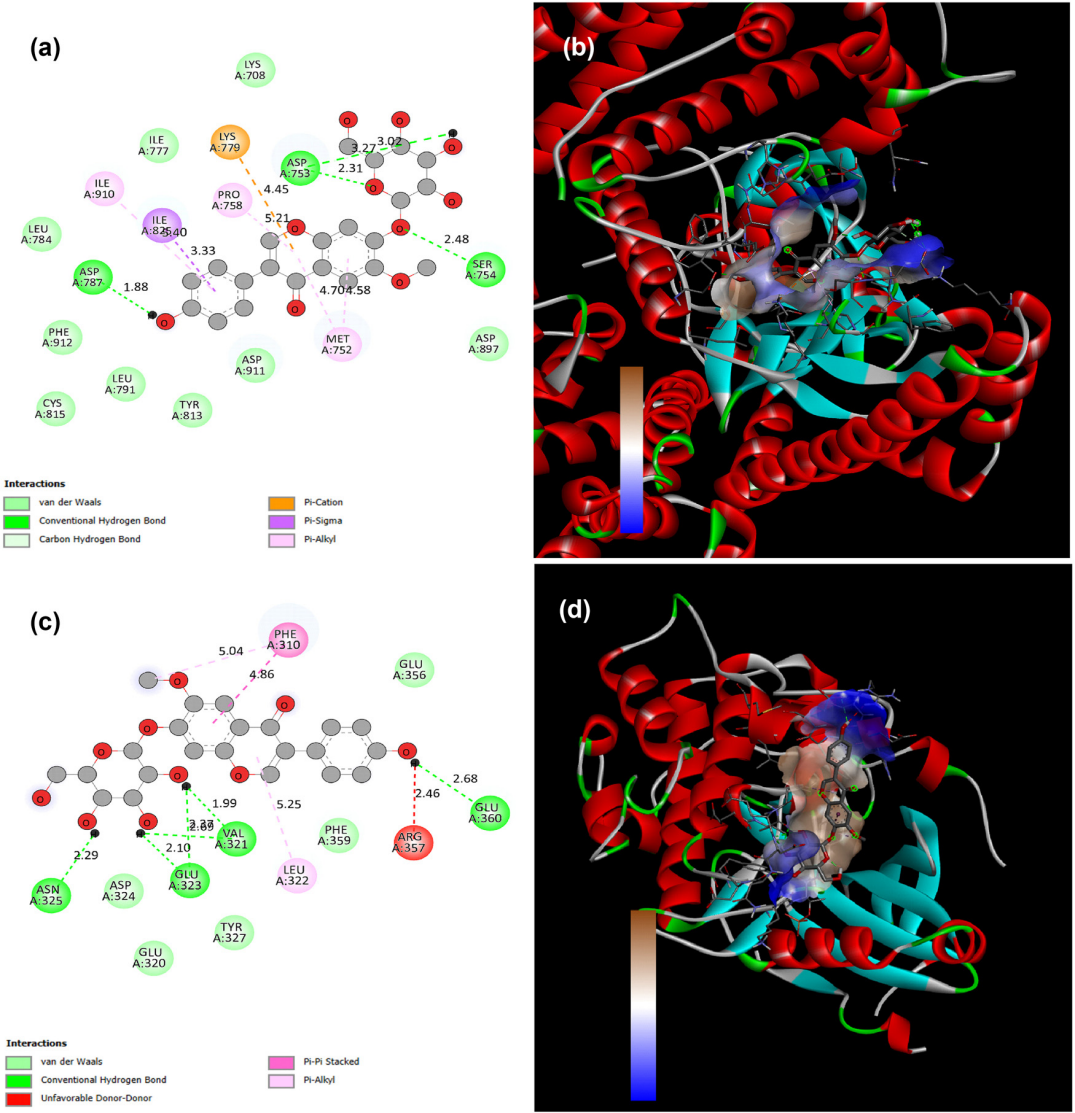
The two- and three-dimensional interactions between GCN compounds and the key target proteins P13K and Akt were analysed. Specifically, (a) and (b) interactions of GCN with P13K two- and three-dimensional structure and (c) and (d) interactions of GCN with Akt two- and three-dimensional structure were examined. These interactions were visualized using Biovia Discovery Studio, which produced 2D and 3D structural representations.

By precisely estimating a ligand’s form inside the boundaries of an attachment pocket, docking aims to evaluate the strength of attachment appropriately. To determine their potential mechanism of action, the bioactive compounds GCN were docked with specific proteins, P13K, and Akt to assess their affinity with their binding sites. Among the top-ranking targets (P13K, Akt), the Akt revealed the highest binding affinity with GCN at −7.65 kcal/mol, followed by P13K at −7.03 kcal/mol. Similarly, the Akt displayed the strongest binding affinity with GCN at −4.66 kcal/mol, followed by P13K at −4.04 kcal/mol (Figure 7a–d).

### 3.11 GCN downregulates PI3K/Akt/mTOR signalling in M5-induced HaCaT keratinocytes

**Figure 8:**
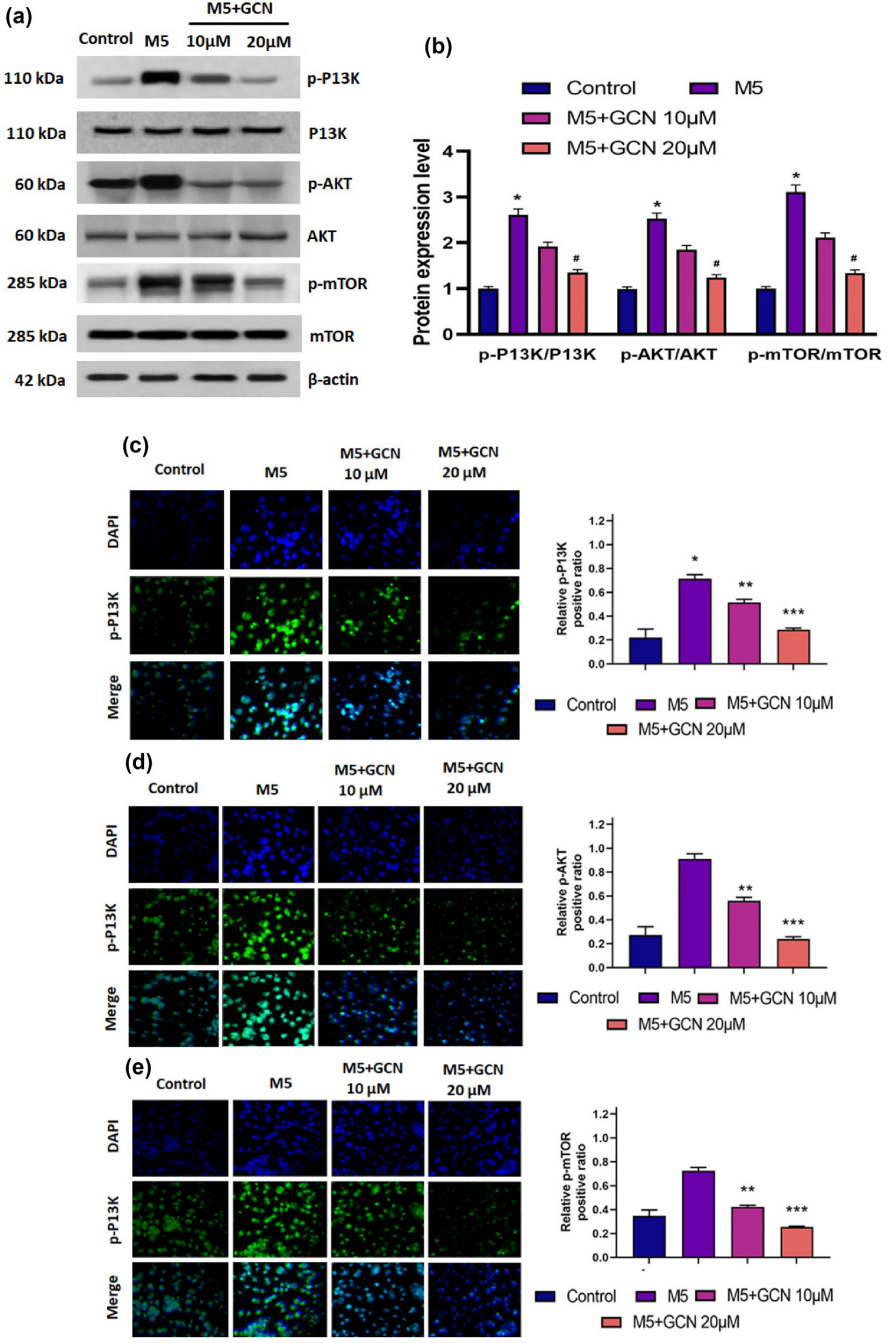
GCN inhibits the PI3K/Akt/mTOR signalling pathway in M5-stimulated HaCaT cells. (a) and (b) Western blot analysis of phosphorylated and total PI3K, Akt, and mTOR in HaCaT cells following treatment with M5 and GCN (10 and 20 µM). (c–e) Immunofluorescence staining for p-PI3K, p-Akt, and p-mTOR, respectively. DAPI stains the nuclei (blue), and phosphorylated proteins are shown in green. Bar graphs show quantitative analysis of fluorescence intensity. Data represent mean ± SE. *p < 0.05, **p < 0.01, **p < 0.001 vs M5-treated group.

Using Western blotting, we examined the PI3K/Akt signalling systems to better understand the molecular processes behind the anti-proliferative and pro-apoptotic impacts of GCN in HaCaT cells. Moreover, M5 activation in keratinocytes was found to be associated with excessive expression of PI3K, Akt, and mTOR. It was discovered that the groups (the untreated and GCN-treated groups) had comparable levels of PI3K, Akt, and mTOR expression. Furthermore, concentration reduction was influenced by the phosphorylated proteins p-PI3K, p-Akt, and p-mTOR. These findings suggested that GCN’s pro-apoptotic and anti-proliferative activities on HaCaT cells contributed to the inhibition of the PI3K/Akt/mTOR signalling pathway. Additionally, GCN treatment led to a significant reduction in the protein expression of phosphorylation of PI3K, Akt, and mTOR, according to immunofluorescence assessment (Figure 8d–f). This suggests that GCN downregulates PI3K/Akt/mTOR signalling in a transcription-independent mechanism. GCN may target PI3K/Akt to produce its effects, as evidenced by the discovery that residues in PI3K/Akt could be linked to GCN by the use of computer-simulated docking molecules (Figure 7). In M5-treated HaCaT cells, our observations suggested that GCN may target PI3K/Akt to inhibit PI3K/Akt/mTOR signalling.

## 4 Discussion

Preserving good skin condition requires an effective skin barrier, and in recent decades, it has been shown that a variety of botanical medicines provide therapeutic potential for several skin barrier problems [33,34,35]. Based on prior evidence linking oxidative stress and PI3K/Akt signalling to keratinocyte dysfunction in psoriasis, we hypothesized that GCN might exert therapeutic effects by restoring redox balance, inducing apoptosis, and attenuating this key pathway. The antioxidant effect of GCN observed in our study is consistent with prior reports suggesting that isoflavones like daidzein and genistein restore redox balance and protect against oxidative damage in skin cells under inflammatory stress [36,37]. Psoriasis constitutes a common papulosquamous skin condition that can lead to cartilage pathology, psychiatric problems, and skin complaints. Development of new pharmacological agents is therefore needed to lessen the disturbance of the skin barrier linked to psoriasis. These pathophysiological disruptions were the foundation for our hypothesis that GCN could restore epidermal homeostasis by inducing apoptosis and reducing inflammatory signalling in keratinocytes. Numerous cells influence psoriasis, a complicated inflammatory skin condition [38]. One kind of resident skin cell that is capable of contributing to and being affected by psoriasis is the keratinocyte. Skin homeostasis depends on the equilibrium involving keratinocyte growth and apoptosis. Skin equilibrium is disrupted in psoriatic lesions. Apoptosis of keratinocytes is regularly found to be decreased, therefore, invariably results in hyperproliferation [39,40]. To maintain and intensify the inflammatory reaction, hyperproliferative keratinocytes generate a significant quantity of cytokines [41,42,43]. GCN may lessen the hyperproliferation of keratinocytes along with the severe inflammatory process that results from this proliferation. GCN thus becomes a possible treatment option for psoriasis.

Numerous *in vitro* cell culture variants that resemble psoriasis have been created recently. These efforts included using imiquimod, INF-γ, or cytokines linked to psoriasis and their combination to stimulate the HaCaT cell line. Among these is the model that uses IL-17A, IL-22, oncostatin-M, IL-1α, and TNF-α to stimulate the HaCaT culture simultaneously [44]. In addition to keratinocyte hyperproliferation and pro-inflammatory response, this M5 stimulation model also exhibits suppression of keratinocyte variation and elevated expression of biomarkers associated with psoriasis [45]. To evaluate the therapeutic relevance of GCN in this context, we used a well-established M5 cytokine stimulation model of HaCaT cells that mimics psoriatic phenotypes. A notable influence of influenced by concentration reduction in the flow of M5-stimulated cytokines into the culture medium was the hallmark of GCN’s anti-inflammatory action in this cell model. Alongside this impact, the hyperactive proliferative phenotype induced by the M5 cytokine mix was normalized, and HaCaT apoptosis increased in response to GCN. The impacts of GCN on the cell phenotype changes in the very pro-psoriatic milieu caused by M5 activation have never been examined before, as far as we are aware.

The natural substance melatonin and its metabolites cause stimulated keratinocytes to become more resistant to apoptosis [46]. This has made a pro-apoptotic approach to psoriasis treatment a viable alternative [44]. For instance, sunitinib causes keratinocytes to undergo apoptosis, which reduces imiquimod-induced inflammation that resembles psoriasis [47]. Additionally, via the fabrication of ROS, erianin demonstrated pro-apoptotic and anti-proliferative effects on keratinocytes, downregulating the Akt/mTOR signalling cascades and upregulating the JNK/c-Jun [48]. According to these studies, pro-apoptosis in psoriasis may have positive results. Previous research has emphasized the therapeutic benefit of promoting keratinocyte apoptosis in psoriasis, with compounds like erianin and sunitinib demonstrating efficacy via ROS-mediated and PI3K/Akt-dependent mechanisms [14,47]. Specifically, keratinocytes were stimulated with M5 to simulate their hyperproliferation condition. The cells were subsequently administered GCN, which resulted in a progressive dose-related reduction in cell viability, and it significantly decreased the expression of KRT6, a marker of excessive rate in psoriasis. After that, we looked into how GCN affected the induction of apoptosis. We discovered that the cells’ morphology had changed significantly, exhibiting apoptotic characteristics as nuclear disintegration and intracellular shrinkage, which were visible through phase-contrast imaging and AO/EtBr and DAPI fluorescent staining. GCN demonstrated anti-proliferative effects on HaCaT cells, according to the current study. This could be because GCN caused HaCaT cells to undergo apoptosis. These findings provide cellular-level evidence for GCN’s therapeutic potential in restoring the balance between keratinocyte proliferation and apoptosis in psoriatic conditions.

As suggested in our hypothesis, oxidative stress plays a critical role in psoriasis, and modulation of ROS may influence disease progression. There is evidence linking the aetiology of psoriasis to instabilities in the oxidation and reduction system [8]. ROS, however, has the potential to regulate chronic autoimmune inflammation [49]. Mitochondrial dysfunction and ΔΨ*m* collapse simultaneously as a result of increased ROS production. Conversely, the buildup of intracellular ROS inhibits cell division by stopping cell cycle stages [50]. Lack of ROS may significantly worsen mannan-induced dermatitis and arthritis, whereas increased ROS production may lessen these inflammatory conditions [51,52]. The study mentioned above demonstrates how ROS can help psoriasis. GCN causes human gastric cancer cells to undergo ROS-dependent apoptosis via the NF-κB pathway [53]. However, as of yet, there is currently no convincing proof linking GCN to ROS regulation in psoriasis. Using DCFDA, JC-1, and PI stain-based cell division analysis, the impact of GCN on the redox status of HaCaT cells with M5 was measured. According to our findings, GCN administration considerably raises the ROS levels in M5-stimulated HaCaT cells. By improving regulatory T cell activity, ROS has recently been demonstrated to mitigate IMQ-induced psoriasis eczema [54]. All things considered; it was suggested that GCN-persuaded ROS in HaCaT cells could potentially be helpful in the therapy of psoriasis. Additionally, JC-1 stain illuminates orange-red and produces J aggregates when it integrates into competent mitochondria. JC-1 will continue to be J-monomers and show glowing green when ΔΨ*m* falls. As the depolarizing process of the membranes of the mitochondria increased, we saw an influence on the concentration evaporation of ΔΨ*m*. The molecular characteristics of apoptosis include single-strand breakage events and the creation of smaller base-pair molecules [55,56]. While the present study provides mechanistic insights into the anti-inflammatory and pro-apoptotic effects of GCN in a psoriatic keratinocyte model, one methodological limitation is the use of the MTT assay for cell viability assessment. Although newer alternatives such as CCK-8 offer improved sensitivity and lower cytotoxicity, MTT remains a widely accepted endpoint assay for fixed-time viability evaluation. The duration of MTT exposure was minimized to avoid prolonged cytotoxicity, and viability results were further validated using complementary assays, including apoptosis analysis and cell cycle profiling. Future studies may consider employing more sensitive assays to enhance translational relevance. Additionally, the impact on the proportion of cell cycle phases as a percentage was examined using flow cytometry. Indicating apoptosis due to internucleosomal DNA disintegration, GCN administration dramatically raised the percentage of the sub-G1 phase group at 10 and 20 μM in comparison to untreated controls, according to the cell cycle analysis results.

The pathophysiology of psoriasis is characterized by aberrant keratinocyte death and uncontrolled proliferation [57,58]. In the current investigation, GCN-treated HaCaT cells exhibited increased early-stage and late-stage apoptosis according to concentration. These findings showed that GCN may considerably enhance the aberrant psoriatic keratinocyte development dynamics. All things considered, the current research indicates that GCN works by strongly inducing apoptosis in M5-induced epidermal keratinocytes.

The PI3K–Akt signalling system was found to be significantly inhibited in response to ROS-induced apoptosis [59]. Using ROS-mediated suppression of the PI3K–Akt signalling process, many cytotoxic drugs have pharmacological effects [60–62]. It has been suggested that the skin cells in psoriasis lesions had hyperactivated PI3K–Akt signalling, a critical pathway that regulates survival signals [11,63–65]. This phosphorylated the resulting downstream proteins targeted FOXO and mTOR, which then in turn promoted the proliferation of keratinocytes [20,66]. According to reasonable reasoning, hindering the PI3K/Akt signalling pathway may be a potential anti-psoriatic treatment [67–69]. It is unknown how GCN affects keratinocytes’ PI3K/Akt/mTOR cascade regulation.

Given the established link between oxidative stress and PI3K/Akt signalling, we explored whether GCN could regulate this cascade in psoriatic keratinocytes. Our findings align with previous studies indicating that targeting the PI3K/Akt pathway can effectively reduce keratinocyte proliferation and cytokine release in psoriatic models, supporting its therapeutic relevance [65,70]. Thus, inhibiting PI3K/Akt/mTOR signalling was a suitable approach to psoriasis therapy. Our findings showed that GCN administration significantly reduced PI3K, Akt, and mTOR phosphorylation, which is in line with other studies. Similar to our findings, previous studies have shown that natural flavonoids such as apigenin and myricetin can attenuate keratinocyte hyperproliferation and inflammatory responses via downregulation of the PI3K/Akt pathway [71,72]. Thus, we deduced that GCN n could function by attaching itself to PI3K and Akt to prevent PI3K/Akt from interacting with its intended substrate DNA. All of the results showed that GCN may function in M5-stimulated HaCaT cells via regulating PI3K/Akt signalling.

In summary, this study provides the first evidence that GCN exerts its anti-psoriatic effects through a dual mechanism involving ROS-induced apoptosis and downregulation of PI3K/Akt/mTOR signalling in M5-stimulated HaCaT keratinocytes. These findings are in direct alignment with the study’s initial hypothesis and highlight the therapeutic potential of GCN for inflammatory skin disorders such as psoriasis. Although our findings demonstrate that GCN treatment increases ROS levels and downregulates key phosphorylated components of the PI3K/Akt/mTOR pathway in HaCaT cells, we acknowledge that a direct causal relationship between ROS modulation and PI3K/Akt signalling was not definitively established. Therefore, future studies employing pathway-specific rescue experiments, such as the use of LY294002, a selective PI3K inhibitor, are warranted to confirm the mechanistic link and further validate the specificity of the GCN-mediated effects on this signalling axis.

## 5 Conclusion

Despite the fact that existences are several psoriasis therapy options, currently, there is always a requirement for novel medications with no adverse reactions and great effectiveness. To the greatest extent of our knowledge, we are the first to demonstrate that GCN has strong anti-inflammatory and anti-proliferative properties that suppress epidermal hyperplasia. GCN can, in summary, reduce the growth and inflammation of HaCaT keratinocytes brought on by M5 cytokines. ROS production and the PI3K–Akt signalling pathway’s subsequent suppression are linked to GCN-mediated HaCaT keratinocyte mortality. Our results point to GCN as a possible pharmaceutical approach to psoriasis therapy; one of its benefits is its biological inflexibility and, therefore, may make it more relevant to psoriasis despite fewer side effects.
